# Sexual healthcare professionals’ views on the rapid provision of remote services at the beginning of COVID-19 pandemic: A mixed-methods study

**DOI:** 10.1177/09564624211023018

**Published:** 2021-06-09

**Authors:** Alexandria Lunt, Carrie Llewellyn, Jake Bayley, Tom Nadarzynski

**Affiliations:** 1 152127 Brighton and Sussex Medical School, Brighton, UK; 2 9744 Barts Health NHS Trust, London, UK; 3 247209 University of Westminster, London, UK

**Keywords:** Sexual health care, healthcare professionals, digital health care, remote health care, barriers, facilitators, COVID-19

## Abstract

Introduction: The COVID-19 pandemic and social distancing measures forced sexual health services to engage with patients remotely. We aimed to understand perceived barriers and facilitators to the provision of digital sexual health services during the first months of the pandemic. Methods: An online survey and qualitative interviews with UK sexual healthcare professionals recruited online and via snowball sampling were conducted in May–July 2020. Results: Amongst 177 respondents (72% female, 86% White, mean age = 46, SD = 9), most utilised telephone and email as their main communication channels; however, their perceived effectiveness varied (94% and 66%, respectively). Most agreed that staff needed additional training (89%), the available technology was not adequate (66%) and health professionals were hesitant to provide online consultations (46%). They had positive attitudes towards digitalisation, improving service quality and cost-effectiveness but were concerned about exacerbating health inequalities. Discussion: The study identifies a need for clear guidelines and training around the use of digital tools as well as a demand for investment in hardware and software required for the provision of remote services. Future research needs to explore the acceptability, safety and effectiveness of various digital tools to narrow health inequalities in sexual health service users.

## Introduction

Between March and May 2020, a UK nationwide lockdown was put into place to reduce excess hospitalisation of patients due to COVID-19, while most outpatient and primary care services restricted face-to-face access, recording a significant decline in attendance and overall healthcare utilisation.^1–2^ Patients were advised to use alternative remote channels of communication, in particular telephone or video consultations with a range of tools such as emails, text message applications, digital leaflets and web chats.^3^ Such a rapid adaptation of digital technologies during the first lockdown had a significant influence on the delivery of services and community-based programmes, often lacking a regulatory framework.^4^ Healthcare professionals had to respond to the rapid provision of innovation to ensure service continuity. However, these were proceeded without contextual guidelines, best clinic practice examples, audits and ongoing evaluations to ensure equitable access and quality of care.

The utilisation of sexual and reproductive health services (SRHS) was also substantially reduced, as demonstrated by an 80% decrease in PEP prescription in a London clinic,^5^ a 78% decrease in Madrid,^6^ and 66% in Melbourne.^7^ This reduction could be associated with a general decrease in sexual activity during this period^8^ but also a possibility of constrained access for asymptomatic cases.^9^ Public Health England reported an overall 13% reduction in consultations, with a 20% increase in digital consultations during the first lockdown accelerating the provision of digital sexual health services (DSHS).^10^ Sexual healthcare professionals (SHPs) were required to utilise digital platforms for remote consultations with little evidence for their effectiveness, safety and acceptability to patients, with some advocating for sexual abstinence.^11^ This led to concerns about exacerbating societal health inequalities due to limited access to technology, lower digital literacy and access to private and safe spaces for intimate conversations across patient groups.^12–13^ Equally, little is known about SHPs’ motivation and capabilities with conducting digital consultations. This study aimed to assess the attitudes of SHPs towards the rapid digitalisation of SRHS in the United Kingdom at the early stage of the COVID-19 pandemic. The objective was to identify the barriers and facilitators for the provision of DSHS to inform service development.

## Methods

### Design

This was a mixed-methods study incorporating an online cross-sectional survey with follow-up telephone interviews to understand the depth and range of views on the provision of DSHS during the first months of the COVID-19 pandemic. The study was approved by the University of Westminster Research Ethics Committee (ref: ETH1920-0979).

### Participants and recruitment

We focussed on health professionals working in SRHS, that is, doctors, nurses and health advisors actively working in clinical practice in the United Kingdom. Between May and July 2020, an online study advert was distributed through Twitter and newsletters of professional organisations relevant to SRHS (i.e. the British Association for Sexual Health and HIV). Tailored invitation emails were also sent out individually to individual members and key sexual health specialists in the United Kingdom with a request to distribute the study advert within their professional networks. Recruitment utilised online snowball and convenience sampling approaches to gathering as many responses as possible. The response rate was not recorded due to the nature of snowball sampling. Participation was voluntary and no incentive was offered. Upon survey completion, participants could provide their contact details to arrange a follow-up interview. Opportunity sampling was obtained by contacting all interested in taking part.

### Measurements and procedure

Upon clicking on the study advert, participants were directed to the online Qualtrics survey which consisted of eight questions and four scales. Participants were asked demographic questions (i.e. gender, age, ethnicity, country of professional practice and professional role) and whether their role was affected by the COVID-19 pandemic. Next, they were shown a list of digital and remote communication channels, such as telephone, email, social media, web chat or phone applications, and asked to indicate which they used in contact with patients. Following this, questions explored the perceived effectiveness of these communication channels, using 5 options ranging from ‘very ineffective’ to ‘very effective’. Afterwards, a nine-item scale explored SHPs’ experiences with providing DSHS, with 7-Likert response options ranging from ‘strongly disagree’ to ‘strongly agree’. These items assessed views on staff training, digital equipment and software, DSHS guidelines and access to IT support. An additional nine-item scale explored attitudes towards the rapid digitalisation of services due to COVID-19, assessing perceived quality, safety and confidentiality of DSHS, perceived level of skills and knowledge regarding digital technologies used in SRHS and the potential impact on health inequalities. The survey took approximately 12 min to complete.

The subsequent follow-up interviews used a topic guide to further explore barriers and facilitators to the provision of DSHS during the COVID-19 pandemic (March–June 2020). They aimed to investigate the lived experiences of SHPs, the impact on SRHS and the usage of technology within the context of COVID-19. The interviews were conducted by AL via telephone or Skype lasting approximately 30 min. All were audio-recorded and transcribed verbatim.

### Data analysis

Descriptive analysis of qualitative data was undertaken using SPSS. Percentages and simple statistical tests (i.e. mean, median, range and standard deviation) were performed and results were presented graphically using column charts. The perceived effectiveness and ineffectiveness of each communication channel was considered alongside their actual usage. Furthermore, the percentages of SHPs agreeing and disagreeing with the attitudinal and experiential questions were considered.

The qualitative data were analysed thematically which involved familiarisation with the data in the written transcripts and the identification of patterns in participants’ responses, in line with the approach recommended by Braun and Clarke (2006).^14^ Microsoft Excel software was used to organise data into themes and subthemes with corresponding quotes. The analysis formulating codes, themes and subthemes was conducted by AL and validated by TN in terms of consistency, coherence and applicability.

## Results

### Attitudinal survey

**Figure 1.**
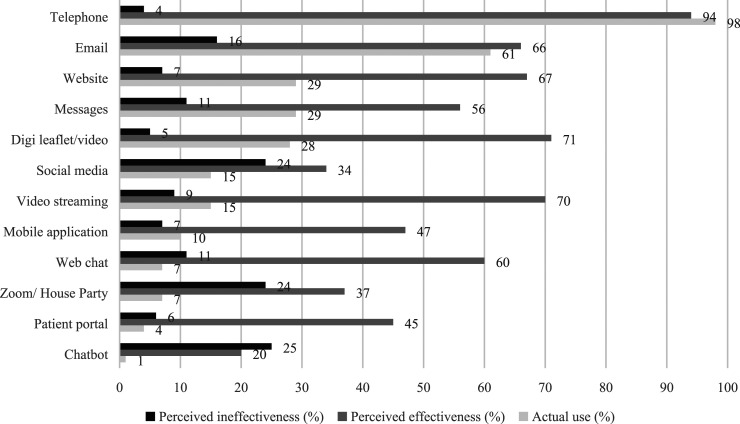
Sexual healthcare professional actual usage, perceived effectiveness and ineffectiveness of different digital communication channels.

In total, 177 SHPs (mean age = 46, SD = 9.7; 72% women; 86% white) completed the survey (Table 1). The majority were located in England (82%), with 46% working as a doctor, 31% as a nurse and 23% as an ‘other’ SHP. Most participants utilised telephone (98%) and email (61%) for communication with service users (Figure 1). About a third reported using message exchange systems such as WhatsApp (29%), websites (29%) and digital leaflets (28%). Social media (15%), video-streaming platforms (15%) and mobile phone applications (10%) were used by a small number of SHPs. Chatbots or virtual assistants (1%) were the least utilised communication method. Telephone consultations (94%), video-streaming platforms such as Skype (70%), emails (66%), digital leaflets (71%), web/live chat (60%) and message exchange platforms (56%) were seen as most effective. Social media (24%) and chatbots (25%) were seen as ineffective.

**Table 1.** Participant socio-demographic characteristics.

Variables	N (%) or (mean, SD)	
(Survey) N = 177		
Gender		
Male	47	(26.6)
Female	128	(72.3)
Non-binary	1	(<1)
Ethnicity		
White	153	(86.4)
Black	4	(2.3)
Asian	13	(7.3)
Mixed	6	(3.4)
Arab	1	(<1)
Age	N = 169	(46.4, 9.7)
Country where practising		
Scotland	9	(5.1)
England	145	(81.9)
Wales	20	(11.3)
Northern Ireland	3	(1.7)
Role		
Doctor	81	(45.8)
Specialist doctor	23	(13)
Consultant	58	(32.8)
Nurse	54	(30.5)
Nurse band 8	9	(5.1)
Nurse band 7	13	(7.3)
Nurse band 5/6	32	(18.1)
Health advisor	20	(11.3)
Healthcare assistant	3	(1.7)
Healthcare support worker	3	(1.7)
Service or departmental manager	2	(1.1)
Psychologist	3	(1.7)
Other (e.g. commissioner, health promotion, sexual health youth worker)	8	(4.5)
If COVID-19 has affected their role		
Yes	64	(36.2)
No	112	(63.3)
(Interviews) N = 24		
Gender		
Male	11	(45.8)
Female	13	(54.2)
Age	N = 24	(51, 10.9)
Country of professional practice		
England	18	(75)
Wales	4	(16.7)
Scotland	2	(8.3)
Role		
Doctor	13	(54.2)
Consultant	10	(41.7)
Specialist doctor	3	(12.5)
Nurse	7	(29.2)
Nurse band 8	2	(8.3)
Nurse band 7	2	(8.3)
Nurse band 5/6	3	(12.5)
Health advisor	2	(8.3)
Psychologist	1	(4.2)
Health promoter	1	(4.2)
Ethnicity		
White	20	(83.3)
Black	1	(4.2)
Asian	2	(8.3)
Mixed	1	(4.2)

**Figure 2.**
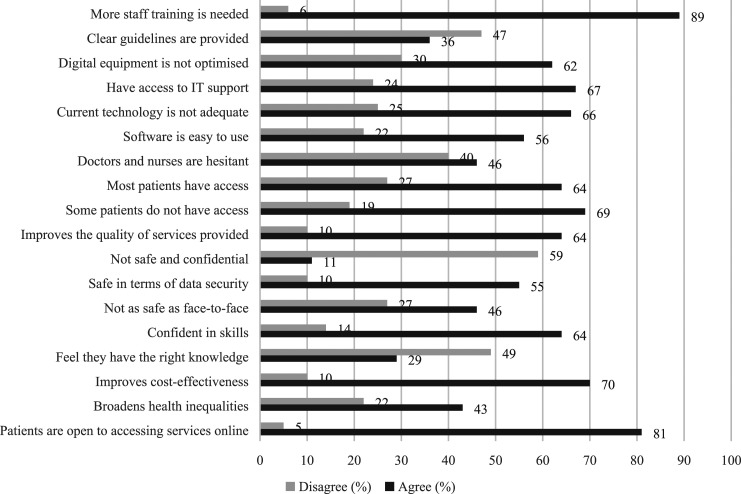
Experiences and attitudes of SHP regarding DSHS in the early stages of the pandemic. SHP: Sexual healthcare professionals; DSHS: digital sexual health services.

As seen in Figure 2, most participants reported the need for staff training (89%) and clearer guidelines (47%) around the use of DSHS. Despite having access to IT support (67%), the majority agreed that their digital equipment was not optimised (62%), with inadequate technology (66%). Around half of SHPs thought that most doctors and nurses were hesitant to provide online consultations (46%), with about a fifth being concerned about patient access to digital services (19%). In general, SHPs had positive attitudes towards the provision of DSHS (69%), with the majority believing that they improved service quality (64%) and cost-effectiveness (70%), and that they were acceptable to service users (81%). However, only half thought DSHS were safe in terms of data security (55%), and a third reported they had the knowledge (29%) and skills (64%) needed to provide DSHS effectively. Just under half were concerned that DSHS may broaden health inequalities (43%).

### Qualitative interviews

Twenty-four survey respondents (age range: 31–76, 54% women, 83% white, 54% doctors) were interviewed (Table 1). Three themes were identified concerning the impact of COVID-19 on services, as well as the barriers and facilitators of the provision of DSHS (Table 2).

**Table 2.** Qualitative themes and exemplar quotes from interviews with SHPs.

Themes (subthemes)	Exemplar quotes
Barriers: Access barriers to the provision of online sexual healthcare services	
(Concerns of disenfranchising certain patients)	“I suspect that we are disenfranchising quite a lot of patients, in other words… the marginalised patients, people with mental health... err English as a second language, the poor, you know, all those sorts of people, I presume, are far less likely to use these sorts of services. Yes, I think they have created barriers.” (Consultant)
	“… but the issue they’ve got is that the people trying to access that are those that are the most vulnerable. So, they might not actually have the right equipment to access it.” (Nurse band 8)
(Insufficient HCP resources)	“… there’s always some sort of financial or operational crisis and managers and people, and therefore the clinical teams are unable to innovate. We’re just not able to innovate and say, ‘let’s spend some money on electronic platforms to sort this problem.’” (Consultant)
	“From a service point of view, our restriction is our IT equipment… there’s no microphones, there’s no cameras, erm so that- the biggest drawback is actually the equipment that we’ve actually got doesn’t support a lot of the things that we’d like to do.” (Nurse band 8)
Barriers: Communication barriers regarding the provision of online sexual healthcare services	
(Hindered rapport)	“… when you have someone in clinic with you on a face-to-face, they tend to divulge more information to you. Erm so more- for me, it’s more looking at the safeguarding aspects erm and about that person feeling comfortable opening up to you. Although we ask the questions I- you don’t-, when you have them in the room you tend to have that bit of chit chat and a bit of a rapport…” (Nurse band 7)
	“With sexual health, because the patients that we generally see- we see them for the first- the first time every time, so there’s very little continuity… so the rapport is even harder to get on the telephone or the video.” (Consultant)
	“Of course, it cannot be a replacement for face to face, and err sexual health is different from any other speciality. We deal with a lot of emotions, and high stress, and people are really concerned. It’s not always the erm the- the physical part of it, or the erm- there is a lot of issues with it that we need to do, a lot of counselling…” (Specialist doctor)
(Lower disclosure)	“… are they in a place where they can- where nobody else is going to be able to hear them? Whereas if they’re in a clinical room, they know it’s a safe space to talk.” (Nurse band 7)
	“… A lot of the time you can pick up queues when you’re sitting next to somebody. You know, they’ll smile when you go ‘right, come on, tell me more about that’ and you’re just missing all that to be fair. That- that sort of visual stuff, I think you miss. And it’s easier to lie to somebody when you’re not looking at them, isn’t it?” (Psychologist)
Barriers: Security concerns regarding the provision of online sexual healthcare services	
(Confidentiality and data security concerns)	“… We’ve had a debate regionally about the mobile phones because of young people… when does a patient stop being a patient? So, if you’re giving advice, how do you record that, in a record?” (Consultant)
	“I think there’s still a level of scepticism about online provision which is partially anxiety, and partially actually quite founded about how easy it is to hack systems or erm you know how people- how levels of people’s education about what cookies are documenting on their computer. Erm we’ve recently started and are just about to launch video consultations which we’ve had to do a lot of information governance work to ensure people that there is absolutely no electronic paper trail.” (Consultant)
(Safeguarding concerns)	“… if I’ve got someone in front of me that I’m able to pull out information form what they’re saying, like there may be abuse going on, as well as other negative things, erm I’m in a position where I can do something about that. Then again, of course, if somebody’s going down the digital avenue, it kind of excludes it…” (Nurse band 5/6)
	“I think when we’re looking at the vulnerabilities aspect and safeguarding, I think those opportunities will be missed by video rather than having a face-to-face.” (Nurse band 7)
Facilitators: Online provision of sexual healthcare facilitates patient access	
(Patient access improvements)	“I think generally people are using online platforms to communicate, to use- in every aspects of their lives. And it’s our job to enable it in healthcare for those that want it.” (Consultant)
	“… there is an opposite side of that that is some people won’t engage with sexual health care unless you offer them some form of electronic path- platform. And I think that’s kind of- that’s some young people as well, some vulnerable people who prefer to communicate with us in an electronic way.” (Consultant)
	“I think we can access most of our patients this way, and actually we’re accessing a group of people who possibly we weren’t engaging with previously… If we are able to reduce erm the kind of demand on our services, by using electronic erm platforms then we, therefore, have got more capacity to see people who are unable to use electronic platforms, to be able to see those people face-to-face… So, hopefully making parity across the whole board…” (Consultant)
(Perceived patient acceptability)	“… My hypothesis is- was that it’s much more acceptable than having to phone up, book an appointment, having to come and see a clinician face-to-face, erm you know, the kind of constraints that we have around that are kind of a pain for patients.” (Consultant)
	“I think most people that have had it offered have been up for either a telephone consultation or erm a video Near Me consultation, or a bit of both. Erm I suppose anecdotally, more younger people are up for that, and maybe older people are less up for that. I haven’t come across it myself. There’s been quite a broad range of people where it’s been offered, and remote consultations have been acceptable.” (Health advisor)
Facilitators: Online provision of sexual healthcare maximises service quality	
(Improved efficiency and convenience)	“I can definitely say there is obvious efficiency in the way we deliver our service. I can definitely say there is a lot of savings in terms of time, in terms of travel costs, in terms of parking, all the issues that we have been struggling with. All these are sorted.” (Specialist doctor)
	“… if you’re over 18 then you can actually apply for and have a home testing kit erm and- and so that is is to- well, potentially to reduce the numbers of people that are accessing the clinic that are asymptomatic and don’t need to come into a clinic.” (Health promoter)
(Effective communication)	“… she felt more comfortable being in her own space, with her own objects, safe environment, being able to talk about more personal things. And some people…. Actually, find it traumatic coming to a sexual health service. You know, just the thought of infections, just the thought of someone seeing them that they may know, or an ex-partner, you know, being judged.” (Health advisor)
	“I do see people relax at home you know they are showing me their cats or kids or whatever they have just decorated a room and they show it and you sort of there is something in that you can talk and build up a trust. But, its actually very difficult when they’re here and they are coming in with raised blood pressure and then they’ve sat in the waiting room for 2 hours and forgot to bring lunch and you know all sorts of things.” (Consultant)
	“So, the time that you actually spend in the clinic will be much less anyway, so the contact time is less and much more valuable because they’re already well informed.” (Consultant)
(Increased disclosure)	“So, the more sensitive the questions, if you have a disembodied voice asking you the question, you might inch err give them- give the answer.” (Consultant)
	“… Most people find that actually phone calls are much better. They don’t like this video stuff, and I think that’s because it’s easier to share when you’re not looking at somebody. So, if you’re saying, ‘oh actually, you know, I’ve been out of lockdown, I have had unprotected sex with a casual person, that actually that’s easier to do when you’re not looking at somebody.” (Psychologist)
	“… Because you’re cutting down that face to face time, they feel less stigmatised I would say.” (Psychologist)
(Added layer of protection for the vulnerable)	“I do use my work email to communicate with patients. Erm tends to people who are a bit vulnerable and might benefit from an added layer of communication with me personally… for people who are vulnerable, certainly in sexual health and HIV, who need a bit of help, I think it can be really helpful.” (Consultant)
	“… if someone’s kind of seen us with a sexual assault a couple of days ago, nothing- nothing stays, you know, because the patient is still in shock. So, I send them an email a week later, it kind of details the different kind of techniques they can use… so that- that information is often tailored to the experience of the patient and what they’ve described…” (Health advisor)
(Widening service range aids a wider range of patients)	“I think it’s maximising the skills of the staff in the sexual health clinic, by seeing the really complex patients that we should be seeing, and those asymptomatic patients who just want a routine check can be managed very easily and very successfully online.” (Consultant)
	“Personally, I think having a number of different approaches, or ways of contacting or interacting with a patient actually helps, because we can pick the one that is going to be best suited to that particular individual. Erm, you know, that’s the thing. And the level of kind of support that someone needs, varies…” (Health advisor)
	“… we’ve got to be very mindful and we’ve got to keep a panoply of services open–face to face, digital, telephone, video, chatbots, for–to kind of cover all of the people that we possibly could, so that we don’t miss anybody or any groups out.” (Consultant)
Facilitators: HCP attitudes regarding experiences of providing online sexual healthcare	
(Positive experiences with online sexual healthcare)	“I feel that- yeah, I feel that it is equitable and anyone that is diagnosed with an STI has to come into the clinic.” (Consultant)
	“It’s not about bums on seats or numbers, this game is about the- the quality of what we deliver, the human cost and the financial cost to the whole nation, not just our simple department. So, I think it needs to be embraced, and it can be embraced, and it will be good and it will really work well for a lot of patients and now’s the time to make sure that all of our patients are having equitable access to all of our services.” (Consultant)
(Need for NHS engagement with innovation and advancement)	“… we’re gonna be left behind, and particularly in sexual health where we’ve got competition now from the third sector and erm private companies wanting to offer sexual health services. If we want to survive within the industry, if you like, and if we want to continue being industry leaders, that we absolutely should be and have to be, then we need to engage with it.” (Consultant)
(Increases patient responsibility and power)	“… What used to happen is you’d have to take the Mifepristone actually in a clinical setting. So, they’ve changed the law now that you can actually take that at home. So, we don’t want to go back. We want to give more power to women, so I’ll be looking in at how that’s changed and how we can carry on.” (Psychologist)
	“But also, to give themselves some control over how often they test. So, our–our home screen kits have been quite popular.” (Health promoter)
COVID-19: Experiences of digitalisation during COVID-19	
(Instigated change)	“I mean, it’s terrible, but it’s given us the- a fantastic opportunity to say, ‘look, this is fantastic, patients absolutely love it, it cuts down time, we can deliver the service much more efficiently.’” (Specialist doctor)
	“… COVID, has changed a lot of that… we’ve been encouraged to innovate and are able to innovate to be able to communicate with people off- online…” (Consultant)
(Positive HCP experiences)	“It’s been very good, very good. It’s made us really analyse what we’re doing, and it’s made us really want to change what we’re doing. Erm and make things a lot easier all around, for both patients and staff… I think we’ve moved on 10 years.” (Nurse band 5/6)
	“I suppose it’s a new technology, a new way of doing things, and we’ll all become better at it and using these types of platforms in the future. I think we’ve all- as staff, we’ve all taken to these things quite well and I’m quite amazed at the positive side of things.” (Health advisor)
(Negative HCP experiences)	“… We’ve cut contact down to the absolute minimum. The shame is that we’ve also cut down what we provide… So, all the guidance has changed. So, we are running a reduced service.” (Psychologist)
	“… it’s err decimated our service in terms of the number of people we’re able to see face to face, err it’s been maybe 10% face-to-face…” (Consultant)
COVID-19: HCP attitudes regarding the future of their services in light of the pandemic	
(Catalyst for permanent service changes)	“… where we’ve streamlined the consultations around the kind of symptoms and we’re only bringing in certain kind of symptomatic patients, so if that continues post-COVID, then great.” (Health advisor)
	“… they don’t want to reset back to pre-COVID erm times- we want to look forward and learn- and see what we’ve learned from this COVID era. And for sexual health that’s fantastic because we’ve continued to provide a service erm and mostly, how can I put it, mostly positive… And we’ve been able to use our information technology and say ‘right, let’s just break the barriers…” (Consultant)

HCP: healthcare professional; MSM: men who have sex with men.

### COVID-19: Experiences and attitudes regarding service digitalisation

All interviewees reported that COVID-19 instigated almost instantaneous change within their services. They viewed COVID-19 as an opportunity to trial digital technologies that their clinics had been considering before the pandemic. Responses to changes were mostly positive and viewed as an advancement of the services. The pandemic enabled re-evaluation of the utility and usefulness of services that had been mostly offline. Most interviewees remarked that COVID-19 was a catalyst for permanent change. The desire for telemedicine and face-to-face clinics being utilised for varied patient needs was remarked upon because it was seen as a ‘streamlined’ and integrative method of sexual health care. Negative perceptions were viewed particularly concerning the experience of working during COVID-19, a time in which their services had been ‘decimated’ and cut back significantly. Some were concerned about the capacity of digital technologies when services return to pre-pandemic demand.

### Barriers: Access, communication and security concerns

Most participants were concerned about patient access to services, disenfranchising certain service users and increasing health inequalities. This pertained notably to the vulnerable, marginalised and minority groups that may not be able to effectively engage with such services due to their inability to use technology, language barriers, lack of safe space for discussions or other insufficient resources. Some SHPs believed that health professionals were not adequately equipped to offer effective online consultations due to inadequate equipment, unstable network connectivity or outdated software. Financial and operational cuts for sexual health were seen as a major barrier to the implementation of DSHS. Sexual healthcare professionals reported communication barriers, such as a reduced ability to form a rapport with patients or to extract sufficiently detailed information such as their sexual health history, via telemedicine. Digital sexual health services were seen as restrictive in providing reassurance and emotional support which are vital for effective and compassionate health care. Participants were concerned about lower disclosure rates, alongside feelings of being less able to pick up visual cues regarding safeguarding and health issues. Sexual healthcare professionals were concerned about confidentiality and data security when using various communication channels, especially during remote working. This was emphasised when describing the handling of patient information and personal details. Sexual healthcare professionals were unsure about maintaining appropriate boundaries with DSHS.

### Facilitators: SHP attitudes and maximising patient access and service quality

The digitalisation of the NHS service was seen as a positive advancement, with most SHPs perceiving DSHS as highly acceptable for their patients. They were seen as convenient and potentially reaching populations that experience barriers to physically accessing services. Many felt that patients should be able to access services from home or work, where service users may be more relaxed and open to discussing their health. Sexual healthcare professionals thought that the provision of DSHS before COVID-19 increased the efficiency of their services, through better demand management and online triage systems. Digital sexual health services were seen to facilitate more time with services users presenting with symptoms while providing more patient-centred care and sexual health education. The anonymity, or ‘disembodied voice’, present within a telephone consultation was seen as beneficial to patient disclosure, especially when discussing issues of higher perceived stigma. Sexual healthcare professionals felt that providing a panoply of services would likely suit a large range of patients, having a wider choice of consultation methods to suit different patient needs. Several interviewees also noted they felt a need for the NHS to engage with innovation and advancement to provide services that correspond to the generational and societal norms. Two of the interviewees noted that DSHS increased patients’ responsibility for their health, by providing methods of self-management and additional choices.

## Discussion

To our knowledge, it is the first survey examining preparedness for and attitudes towards rapid digitalisation of SRHS in response to the COVID-19 pandemic. It demonstrates positive views on DSHS as well as concerns for safeguarding and increased health inequalities due to limited access to technologies in specific patient groups. Despite the availability of guidelines on the digital transformation of SRHS^15^ in January 2020, a substantial proportion of the sample felt that the support for digitalisation was inadequate, expressing a need for additional training and equipment to provide remote services safely and effectively. Digital sexual health services were seen to increase patient access, improve service quality, aiding STI testing uptake, virtual diagnoses and managing demand or clinical workflow. Most SHPs were receptive to the digitalisation of SRHS; however, there was a strong emphasis on narrowing, rather than widening, health inequalities with the help of technology.

The participants identified important barriers for the successful provision of DSHS on both healthcare provider and recipient levels. The implementation of DSHS could be impeded by insufficient resources, notably outdated hardware, software and poor connectivity with patients. Thus, an investment is required to reduce barriers related to technological deficiency and disparity across SHS in various local authorities and simultaneously ensure the effectiveness and safety of DSHS. It is equally important to ensure that the ‘digitally disengaged’ can still access SHS via several other routes in order to ensure that the digital divide is not furthered, thus exacerbating health inequalities. Sexual healthcare professionals felt they had inadequate knowledge and skills regarding digital technologies, that the added digital layer mediates the efficacy of communication and disclosure, and that there is a lack of ethical consideration, each of which potentially impacts digital patient care. This is in line with previous research highlighting the potential harm of DSHS, considering the sensitive nature of sexual health consultations.^16^ A systematic review of 12 studies on digital competencies amongst healthcare professionals showed that experiences of technology and attitudes towards innovation have an impact on individual motivation to provide online healthcare services.^17^ As such, there is a need for ongoing training, digital education and organisational support to maximise these competencies. Similarly, a qualitative study of 18 healthcare experts in Germany showed that digitalisation of healthcare services was restricted by the absence of interoperability, hesitancy due to insufficient evidence on cost-effectiveness and safety as well as the lack of political will, legislation and financial regulations.^18^ There is a possibility that the lack of familiarity with technology, perceived ease of use, computer self-efficacy and objective usability have influenced hesitancy towards some of the communication channels for sexual health advice.^19,20^ The perceived ineffectiveness of specific platforms for doctor–patient communications could be driven by the lack of familiarity; hence, training aimed at building skills and IT proficiency may alter these perceptions. Our present study identified financial cuts as an important obstacle for healthcare digitalisation. Thus, the transformation needs to be standardised and optimised by self-regulatory bodies overlooking the developmental process and providing incentives for digital solutions. More research is needed to examine the equity, acceptability, reach and cost-effectiveness of digital healthcare services to inform stakeholders about the value of innovation.

Although the mixed-methods design implemented in this study provides a more in-depth understanding of views on SHS digitalisation, several methodological issues exist. The views expressed in this study represent various perspectives on the use of telemedicine in the early stage of the COVID-19 pandemic, and these were likely evolving in line with the investment and training offered to sexual health staff. Due to opportunity sampling methods, an uneven distribution between socio-demographic categories within both datasets occurred. Our snowball sampling recruitment strategy may be associated with self-selection bias when health professionals with well-established views on telemedicine were more likely to participate. There were no participants from Northern Ireland, and there were fewer nurses than can be represented within the national workforce as a whole. Within both arms of the study, there was a skew towards consultants working in England and may not be representative of all perspectives within sexual health workers. There is no standardised questionnaire on attitudes towards digitalisation, and the Cronbach alpha coefficient relating to the survey was only at an acceptable level, indicating lower internal consistency of the measures. Therefore, the mean scores obtained from the Likert scales used to measure SHP attitudes and experiences may not be fully representative of the variables, limiting inferential statistics.

To conclude, the focus should be on a digitally enabled healthcare system, wherein a variety of communication methods are available to suit the patients’ needs, referring asymptomatic and non-complex patients to DSHS, and symptomatic, complex or vulnerable patients to in-clinic services. Digital technology allows for more patient-centred services with specific information being tailored to patients’ skills and characteristics. Thus, national guidelines on digital sexual health should be updated to reflect changes in technologies, user acceptability and various layers of barriers. Future research should explore the motivations and skills for DSHS in order to monitor any provider-level barriers to the provision. There is a need to understand whether there are discrepancies between specific professional roles or UK regions regarding remote services provision. This study offers insights into the baseline acceptability rates of various digital channels and platforms for online engagement with patients. Such a survey could be repeated in the future to assess the change in attitudes towards digital sexual health services and a potential reduction or increase of barriers.
